# SAS6-like protein in *Plasmodium* indicates that conoid-associated apical complex proteins persist in invasive stages within the mosquito vector

**DOI:** 10.1038/srep28604

**Published:** 2016-06-24

**Authors:** Richard J. Wall, Magali Roques, Nicholas J. Katris, Ludek Koreny, Rebecca R. Stanway, Declan Brady, Ross F. Waller, Rita Tewari

**Affiliations:** 1 School of Life Sciences, Queens Medical Centre, University of Nottingham, Nottingham, UK; 2 Department of Biochemistry, University of Cambridge, Cambridge, UK; 3 School of Botany, University of Melbourne, Parkville, Victoria, Australia; 4 Institute of Cell Biology, University of Bern, CH-3012 Bern, Switzerland

## Abstract

The SAS6-like (SAS6L) protein, a truncated paralogue of the ubiquitous basal body/centriole protein SAS6, has been characterised recently as a flagellum protein in trypanosomatids, but associated with the conoid in apicomplexan *Toxoplasma*. The conoid has been suggested to derive from flagella parts, but is thought to have been lost from some apicomplexans including the malaria-causing genus *Plasmodium.* Presence of SAS6L in *Plasmodium*, therefore, suggested a possible role in flagella assembly in male gametes, the only flagellated stage. Here, we have studied the expression and role of SAS6L throughout the *Plasmodium* life cycle using the rodent malaria model *P. berghei*. Contrary to a hypothesised role in flagella, SAS6L was absent during gamete flagellum formation. Instead, SAS6L was restricted to the apical complex in ookinetes and sporozoites, the extracellular invasive stages that develop within the mosquito vector. In these stages SAS6L forms an apical ring, as we show is also the case in *Toxoplasma* tachyzoites. The SAS6L ring was not apparent in blood-stage invasive merozoites, indicating that the apical complex is differentiated between the different invasive forms. Overall this study indicates that a conoid-associated apical complex protein and ring structure is persistent in *Plasmodium* in a stage-specific manner.

The spindle assembly abnormal 6 homolog (SAS6) protein is required for early centriole assembly and coordinated basal body formation in a multitude of eukaryotes including humans^1^. Flagella initiate from this centriole/basal body and typically consist of a 9+2 formation of microtubules. This process is regulated by SAS6 which has a well-defined structure with a globular domain that homodimerises and a long coiled-coil domain. Nine dimers self-assemble into a ring structure with outward radial coiled-coil spokes. This cartwheel shaped structure is required for the establishment of radial symmetry of the microtubules making up the centriole and then flagellum^2^. SAS6 is ubiquitous in flagellated eukaryotes including apicomplexan parasites as reported recently in *Plasmodium berghei*^3^,^4^,^5^. The phylum Apicomplexa contains some 6000 described obligate intracellular parasites, including the causative agent of malaria, *Plasmodium* spp., and other human pathogens *Toxoplasma gondii* and *Cryptosporidium* spp^6^. Flagellated stages in Apicomplexa have been reduced to the male gametes (microgametes) only, with most parasite motility in other cell stages performed using a myosin-driven gliding motility system that spans the plasma membrane^7^. In *Plasmodium*, SAS6 expression is, therefore, restricted to the male gametocyte/gamete, with multiple single dot localisations corresponding with the presence of eight putative centrioles that give rise to eight flagellated microgametes^5^,^8^,^9^,^10^. Gene deletion shows that SAS6 has a role in the formation of viable and motile flagella in male gametes. These mutants also show abnormalities in the proper number of microtubules as well as flagella beating suggesting SAS6 regulates flagella formation and function in microgametes^5^. SAS6 behaviour differs in *Toxoplasma gondii* in that it is present in asexual, non-flagellate cells, associated with the centriole which is persistent in coccidia throughout most cell stages, unlike in *Plasmodium*^3^.

Recently a paralogue of SAS6, SAS6-like (SAS6L), has been investigated in two disparate eukaryotes, *Toxoplasma* and *Trypanosoma*^3^. SAS6L is widespread in eukaryotes although absent from metazoans^3^. This SAS6 paralogue lacks the coiled-coil domains that form the long radial spokes of the SAS6 complex, instead consisting primarily of the protein globular domain that, for SAS6, forms the oligomeric ring of nine dimers. It is not known what conformation or structures SAS6L is capable of forming, however its location has been determined in both the trypanosomatid *Trypanosoma brucei*, that is permanently flagellate, and the apicomplexan *Toxoplasma gondii*. In *T. brucei*, SAS6L localises near SAS6 in the basal body, but at a distinct distal position within the transition zone. Curiously, in *T. gondii*, SAS6L is not located near SAS6 at the centriole, but instead at the apical end of the cell, specifically at the distal region of the conoid within the apical complex. Electron microscopy reveals a pair of preconoidal rings at this position, although the precise location or structure that SAS6L assumes was not determined. Nevertheless, SAS6L was shown to move with the conoid during extrusion of this structure, and remains associated with it after detergent extraction, suggesting an intimate association of SAS6L with the conoid^3^.

The conoid is a conspicuous feature of many apicomplexans and is found at the centre of the apical complex—the defining feature of Apicomplexa and major instrument for host cell invasion. Common to the apical complex- is the apical polar ring, a ring-shaped apical microtubule organising centre from which subpellicular microtubules extend^11^,^12^. These microtubules support the plasma membrane and inner membrane complex (IMC), which together provides the shape and stability of the parasite^13^. Secretory organelles, micronemes and rhoptries, cluster at the cytosolic side of the apical complex, and secrete through the apical polar ring during invasion. The conoid is a collar-shaped structure that sits within the apical polar ring, although separate from it, just beneath the apical portion of the plasma membrane. In taxa such as *T. gondii* it is highly mobile, retracted posterior to the apical polar ring when growing within host cells, but dynamically relocated through and anterior to the apical polar ring during invasion events. Despite this conspicuous behaviour in *T. gondii*, the function of the conoid is not well understood although it is presumed to provide some mechanical role during secretion and invasion^14^. The molecular composition of the conoid is also only superficially known. In *T. gondii* the major structural elements are tightly coiled tubulin filaments^11^,^15^,^16^. Other proteins closely associated with the conoid in *Toxoplasma* include calcium-binding domain proteins (CAM1 and 2), dynein light chain (DLC), centrin 2, RNG2, intra-conoid microtubule associated protein 1 (ICMAP1), myosin H (MyoH), striated fiber assemblins (SFA) and SAS6L^3^,^13^,^17^,^18^,^19^,^20^. It is, however, not known: 1) what the functional contributions of these conoid-associated proteins are; 2) what other proteins contribute to conoid structure and function; and 3) how well conserved conoid composition and function is throughout Apicomplexa.

The conoid is identified broadly in Apicomplexa: in gregarines, coccidian and some hematozoans^21^,^22^,^23^. It shares similarity to apical open-sided collar structures made of microtubules, so-called “pseudoconoids”, in close relatives of Apicomplexa (e.g. *Chromera*, Colpodellids, *Perkinsus*), that are closely associated with the flagellar root apparatus^24^,^25^. Conoid-associated proteins SAS6L and SFA are also typically found in basal bodies or flagellar roots^3^,^26^. These data imply that the conoid is an ancient feature that might have played a central role in the evolution of the apical complex from elements of the flagellum/flagellar root system. *Plasmodium* has been traditionally considered to lack the conoid (it is classified in the Aconoidasida) based on ultrastructural data, particularly for the blood-stage merozoite. This suggests reduction and possible loss of this structure. Nevertheless, *sas6l* is present in *Plasmodium*, raising the question of its role in these parasites. Flagellar formation is unusual in *Plasmodium*, where the axoneme forms in the cytosol before exflagellation, so a role for a different SAS6 version in this process has been suggested^3^.

To examine the localisation and possible function of SAS6L in *Plasmodium*, we investigated SAS6L in the rodent malaria model *P. berghei*. All *Plasmodium* species undergo a complex life cycle in multiple hosts (vertebrates and mosquito) and include different invasive zoite stages that are required to penetrate a variety of cell and tissue types. Use of *P. berghei* has allowed us to examine SAS6L in all stages of this complex life cycle including male microgamete formation, the only flagellated stage. These studies showed that SAS6L is not associated with the basal body of microgamete flagella, but instead, like in *Toxoplasma*, is associated with the apical complex where it forms a ring. Furthermore, this ring is absent in merozoites found in the mammalian host (liver and blood), but present in both ookinetes and sporozoites during the stages that form within the mosquito vector. These data suggest that there are distinct compositions of the apical complex that occur between the different zoite stages of *Plasmodium*, and that conoid-associated proteins are present in *Plasmodium* despite the apparent loss of the tubulin component of this structure.

## Results

### SAS6L expression is primarily limited to ookinete and sporozoite developmental stages

**Figure 1:**
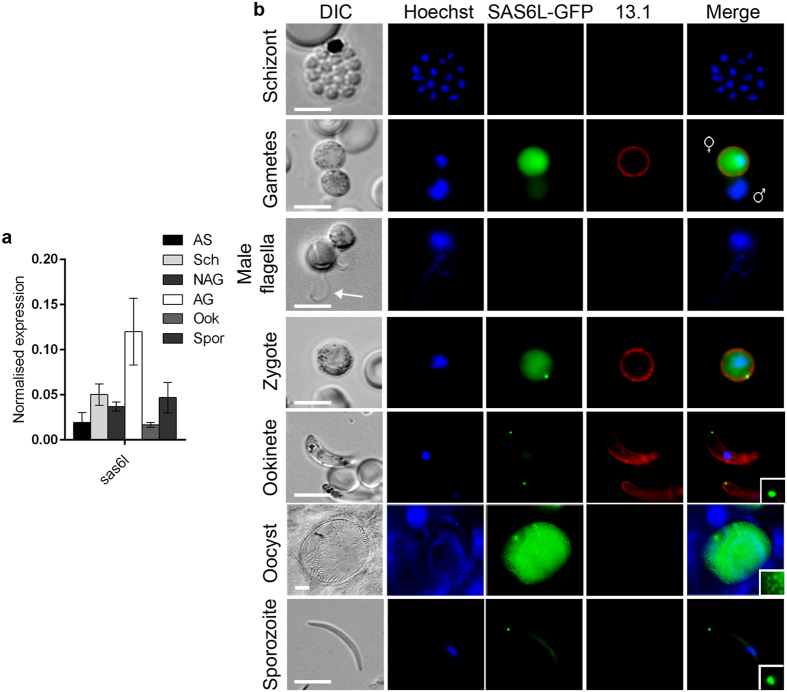
SAS6L gene transcript and protein expression throughout the life cycle in *P.berghei*. (**a**) Transcription of *sas6l* analysed by qRT-PCR, normalised against two endogenous control genes, *hsp70* and *arginine-tRNA synthetase* (Pfaffl method). Each bar is the mean of three biological replicates ± SEM. All asexual blood stages: AS; schizonts: Sch; non-activated gametocytes: NAG; activated gametocytes: AG; ookinete: Ook; 14 dpi oocysts/sporozoites: Spor. (**b**) Live fluorescence detection of SAS6L-GFP in blood-stage schizont, female and male gametes, male gamete with flagella, zygote, ookinetes, oocyst (14 dpi) and sporozoite (21 dpi). 13.1, a cy3-conjugated antibody which recognises P28 on the surface of activated female, zygote, and ookinete (red) was used with the sexual stages. Nuclei were detected using Hoechst 33342 (blue), and the cells were displayed by differential interference contrast (DIC). Merge is the composite of Hoechst, GFP and 13.1 signals. Insets show magnified view of SAS6L-GFP dots. White arrow points to single flagellated male gamete. Scale bars = 5 μm.

To investigate the transcription of *sas6l* during the *Plasmodium* life cycle, we used qRT-PCR and showed *sas6l* RNA is transcribed throughout all parasite stages tested, with strongest expression seen in activated gametocytes (Fig. 1a). This compares well with previous transcript analysis in *P. berghei*^27^. The localisation and expression of SAS6L was investigated by generating a C-terminal GFP fusion protein by single homologous recombination immediately downstream of the *sas6l* locus in *P. berghei* (PBANKA_1414900; Supplementary Fig. S1). Successful integration was confirmed using diagnostic PCR (Supplementary Fig. S1). Western Blot analysis with an anti-GFP antibody showed that the SAS6L-GFP protein in activated gametocytes and ookinetes was the correct size (55 kDa) compared with WT-GFPcon 507 cl1 parasites constitutively expressing GFP (29 kDa)^28^, known henceforth as WT (Supplementary Fig. S1).

Live imaging and anti-GFP immunofluorescence assays (IFA) did not detect any SAS6L-GFP in any blood-stage asexual cells including mature schizonts and invasive merozoites (Fig. 1b, Supplementary Fig. S1). Very faint cytosolic SAS6L-GFP expression was identified using IFA in liver stage mature schizonts containing merozoites, although this signal was not detected in live imaging (Supplementary Fig. S1). During blood-stage transition to the sexual stages, no expression was seen in activated male gametocytes/gametes (Fig. 1b, Supplementary Fig. S1). By contrast, a strong cytosolic localisation was seen in female gametocytes/gametes (Fig. 1b). After fertilization, this cytosolic staining was retained in zygotes along with the appearance of a dot at the periphery of the zygote. The cytosolic staining was progressively lost during later stages of ookinete development however this dot remains localised to the ookinete apical end (Fig. 1b). During sporozoite formation in oocysts at 14 days post-infection (dpi), the SAS6L-GFP signal is, again, faintly cytosolic, with small GFP dots observable corresponding to the ends of individual sporozoites. Isolated individual salivary gland sporozoites confirmed these dots to be present at the cell extremity (Fig. 1b).

### SAS6L locates to an apical ring early during the differentiation of invasive stages within the mosquito vector

**Figure 2:**
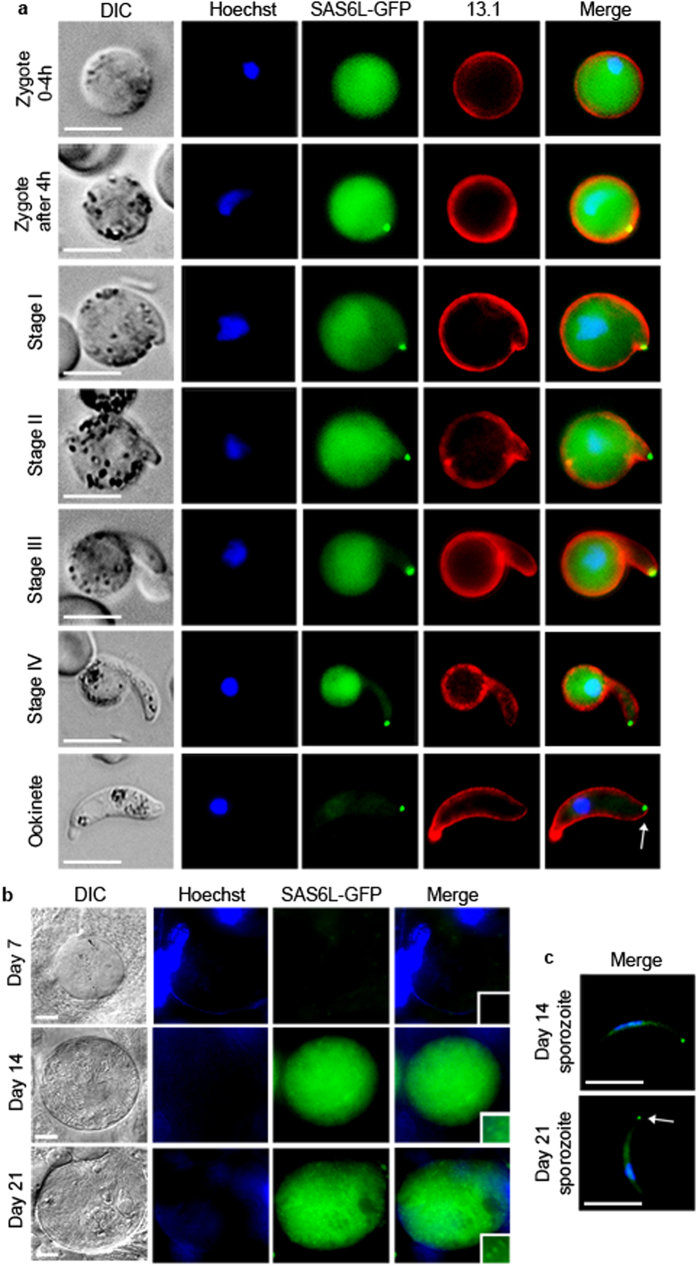
Developmental profile of SAS6L in ookinetes and sporozoites. (**a**) Localisation of SAS6L-GFP throughout the different stages of ookinete development (from early zygote to ookinete stages). 13.1 labels the surface (red) of the sexual stages as for Fig. 1. Nuclei were detected using Hoechst 33342 (blue), and the cells were displayed by differential interference contrast (DIC). Merge is the composite of Hoechst, GFP and 13.1. (**b**) Localisation of SAS6L-GFP throughout the mosquito development (from 7 dpi to 21 dpi). Merge is the composite of Hoechst and GFP. (**c**) Localisation of SAS6L-GFP in isolated 14 and 21 dpi sporozoites. Scale bars = 5 μm.

To further characterise the localisation of SAS6L during the development of invasive mosquito stages we first followed temporal localisation of SAS6L-GFP throughout ookinete development—from zygote to the motile ookinete stage over a 24 h time period. During the first 4 h post-fertilization of female gametes only cytosolic localisation of SAS6L-GFP is seen, however after 4 h a single strong dot appears at the periphery of the zygote (Fig. 2a). As the ookinete develops, this SAS6L-GFP signal marks the point of emergence of the forming zoite, and associates with the apical tip as it extends (Fig. 2a). Cytosolic SAS6L-GFP is apparently excluded from the developing zoite, and this cytosolic signal is ultimately lost as the ookinete matures (Fig. 2a). SAS6L-GFP gametocytes were crossed with gametocytes from an ISP1-mCherry line, a protein specific to the apical cap of the IMC^29^, confirming that the SAS6L dot remains at the cell apex post ookinete maturation (Supplementary Fig. S1).

A similar developmental pattern of SAS6L was observed during sporozoite differentiation in 7, 14 and 21 dpi oocysts (Fig. 2b). Oocysts at 7 dpi showed no SAS6L-GFP expression, but by 14 dpi a cytosolic signal occurs along with individual dots. This signal seems to correlate with the onset of sporogony, with the dot signal increasing in intensity after the sporozoites are fully formed inside the oocyst which persists through to 21 dpi oocysts (Fig. 2b). Isolation of individual 14 and 21 dpi sporozoites revealed that the dot is localised at the apical end of the sporozoite (Fig. 2c).

**Figure 3:**
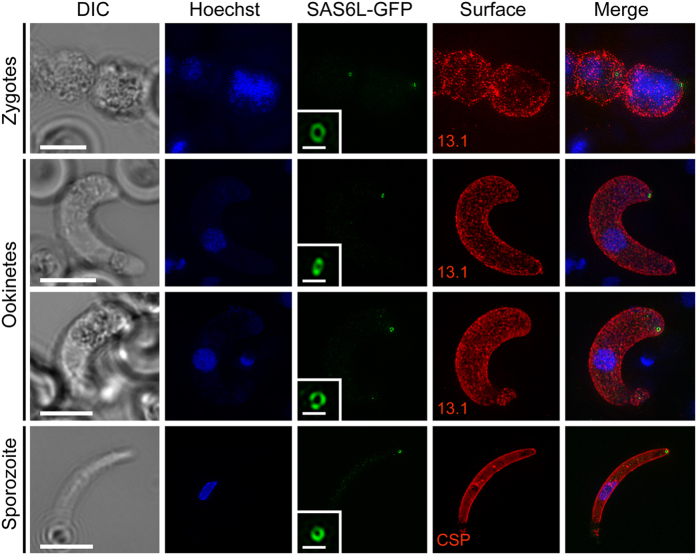
3D-SIM super-resolution images of SAS6L shows a ring structure at the apical end of the ookinete and sporozoite. Images of fixed zygotes, ookinetes, and salivary gland sporozoite at 21 days post-infection expressing SAS6L-GFP (green), co-stained with DAPI (blue), 13.1 (red: zygotes/ookinetes) or CSP (red: sporozoites). Images were selected for the orientation of the apical end of the cell facing the viewer and displaying the ring. Major scale bars = 5 μm. Insets show magnified view of SAS6L ring structures with scale bars = 500 nm.

To better resolve the apical structure labelled by SAS6L-GFP we used 3D-SIM super-resolution microscopy. In all stages this showed that SAS6L locates to a ring structure at the very apical end of the cell (Fig. 3). This includes the SAS6L signal seen at the periphery of zygotes, before ookinete development commences (Fig. 3). These data suggests that SAS6L is either recruited to, or itself forms, a ring-structure during early differentiation of the apical complex in these insect zoite stages.

### *Toxoplasma* SAS6L also forms an apical ring preceded by cytosolic occurrence during nascent conoid formation.

**Figure 4:**
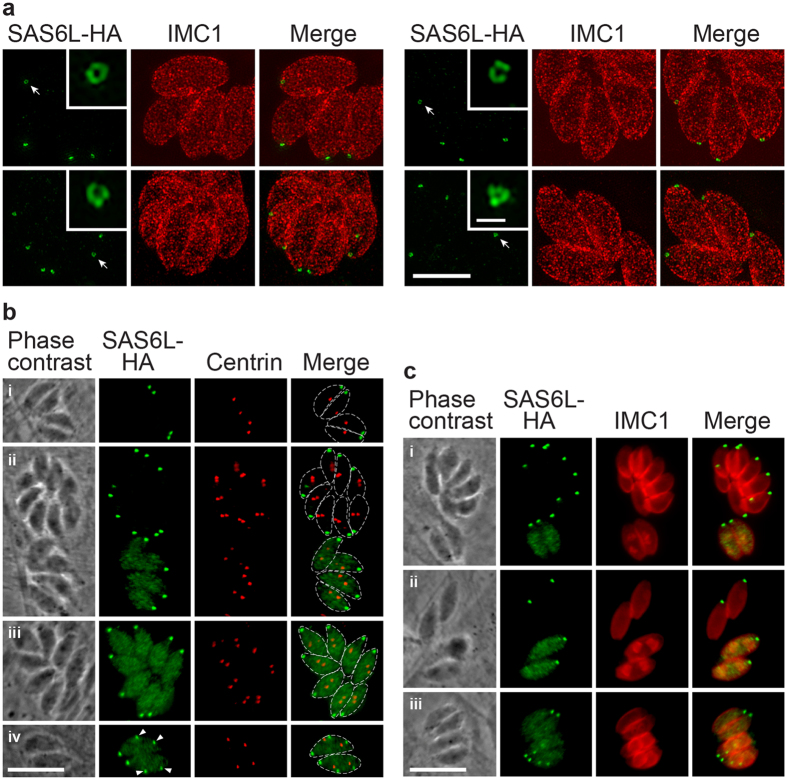
SAS6L location and behaviour throughout the tachyzoite cell cycle in *Toxoplasma gondii*. *T. gondii* expressing endogenous SAS6L with C-terminal fusion to HA-APEX fixed in host vacuoles and stained for HA (green) and either IMC1 (red, **a** and **c**) or centrin (red, **b**) (**a**) 3D-SIM super-resolution images with cell apices facing the viewer showing SAS6L rings (arrows, insets) and apices in profile showing SAS6L flush with the apical IMC. (**b**) Widefield images of parasites at different points of the cell cycle indicated by centrosome number and position (stained for centrin): interphase with single centrosome per cell (i); newly divided centrosomes close together (ii, upper vacuole); divided centrosomes migrated further apart (ii, lower vacuole; and iii); cells with nascent apical complexes (iv, arrowheads). (**c**) Widefield images of parasites showing daughter pellicle formation (stained for IMC1): before daughter pellicle formation (i and ii, upper vacuoles); small pellicle cups (i and ii, lower vacuoles; iii, upper vacuole); mid pellicle development (iii, lower vacuole). Scale bars = (**a**) 5 μm and 500 nm (inset); and (**b**,**c**) 10 μm.

Previous studies showed that SAS6L is attached to the apical end of the conoid in *T. gondii*, but the fine structure formed by SAS6L was not resolved, and its behaviour throughout the cell cycle not described^3^. To visualise SAS6L through the cell cycle in *T. gondii* we performed endogenous tagging of the *sas6l* gene by 3′-replacement with coding sequence for a reporter protein fusion, HA-APEX. 3D-SIM super-resolution IFA microscopy revealed that SAS6L forms a discrete ring at the cell apex, identical in appearance to that seen in *P. berghei* ookinetes and sporozoites (Fig. 4a). This ring occurs at the same plane as the apical IMC in intracellular parasites, consistent with its location at the distal tip of the conoid as previously described^3^. To observe SAS6L location throughout the cell cycle, we counterstained parasites with either: centrin, to visualise centrosome duplication as one of the earliest markers of commencement of cell division; or IMC1, to visualise development of the nascent daughter cell pellicles. When newly duplicated centrosomes were still very close to each other, the only SAS6L signal was associated with the mother cell apical complex (Fig. 4bii), as is the case for interphase cells. As centrosome separation occurred, appearance of SAS6L dispersed throughout the cytosol was seen (Fig. 4bii,iii). This cytosolic signal persisted as IMC1-stained daughter pellicles first appeared, but the major SAS6L puncta remained those of the mother cell apical complexes (Fig. 4ci,ii). As the pellicles elongate SAS6L developed an increasingly bright spot at the end of each daughter cell until it matched the intensity of the mother apical complex (Fig. 4ciii). Over this period, the cytosolic signal steadily diminished until it was no longer seen.

### SAS6L is not essential for any developmental stages of the parasite life cycle

To test for putative functions of SAS6L in *Plasmodium* throughout the full life cycle, we created a null mutant by deletion of the *sas6l* gene using double homologous recombination (Supplementary Fig. S2). Two knockout clones were generated from two independent transfections, ∆*sas6l* cl.2 and ∆*sas6l* cl.3, with successful deletion confirmed by diagnostic PCR showing integration of the *dhfr/ts* cassette and deletion of the *sas6l* gene (Supplementary Fig. S2). Southern blot also confirmed that the gene was successfully deleted (Supplementary Fig. S2).

**Figure 5:**
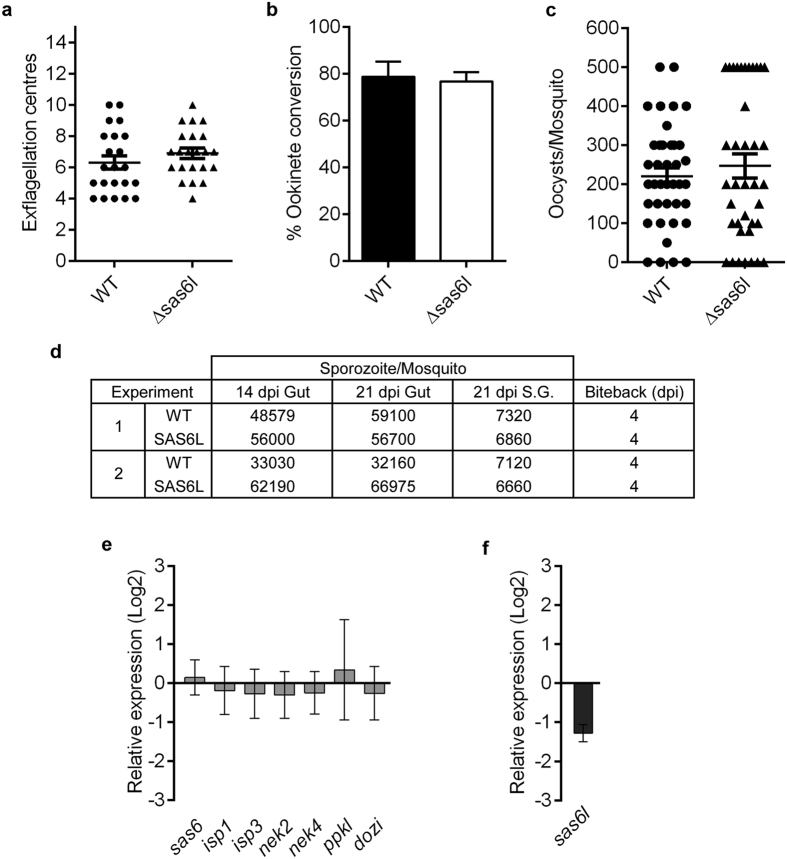
Gene deletion shows that *sas6l* is not essential in parasite development and transmission. (**a**) Microgametogenesis of ∆*sas6l* in comparison to WT measured as the number of exflagellation centres per field. Means ± SEM are shown. n = 22 from three independent experiments (cl. 2 = 2 experiments; cl. 3 = 1 experiment). (**b**) Ookinete conversion as a percentage in ∆*sas6l* and WT lines. Ookinetes were identified using the marker 13.1 and were defined as those cells that successfully differentiated into elongated ‘banana shaped’ ookinetes. Bar is the mean ± SEM. n = 4 (cl. 2 = 3; cl. 3 = 1) independent experiments. (**c**) Oocysts per mosquito gut (14 days post-infection; bar = arithmetic mean ± SEM; n = 38 from three independent experiments. (cl. 2 = 2 experiments; cl. 3 = 1 experiment) of Δ*sas6l* or WT parasite-infected mosquitoes. An infection rate of >80% was observed for both Δ*sas6l* and WT parasites. (**d**) Sporozoites per mosquito for 14 and 21 dpi and bite-back experiments indicating successful transmission of both Δ*sas6l* and WT parasites from mosquito to mouse. Infected mosquitoes were allowed to feed and the mice were monitored for blood stage parasitaemia (days post infection, dpi) indicative of successful liver and blood stage infection. Example of two independent experiments (cl. 2 and cl. 3) are shown. (**e**) Relative expression of *sas6*, *sas6l*, *isp1*, *isp3*, *nek2*, *nek4*, *ppkl* RNA in Δ*sas6l* parasites and (**f**), *sas6l* RNA level in Δ*dozi* mutant parasites compared to WT controls (∆∆Ct method). Mean ± SEM, n = 3 biological replicates from at least two independent experiments. One representative experiment is shown.

Analysis of both Δ*sas6l* clones (cl.2 and cl.3) showed no overt phenotype during *in vitro* microgamete exflagellation (flagella formation) or ookinete conversion when compared with WT control parasites (Fig. 5a,b). When the Δ*sas6l* parasites were fed to female *Anopheles stephensi* mosquitoes, no significant difference in the number of oocysts compared to WT controls was observed (Fig. 5c,d). Finally when Δ*sas6l*-infected mosquitoes were allowed to feed on mice in bite-back experiments, we found that transmission through to blood-stage parasites occurred successfully as for WT parasites (Fig. 5d).

### Deletion of *sas6l* resulted in no differential regulation of related genes

To further characterise Δ*sas6l* parasites and to investigate a possible role for *sas6* in compensating for the absence of *sas6l*, we performed qRT-PCR analysis using Δ*sas6l* activated gametocytes compared with WT (Fig. 5e). We found no change in *sas6* mRNA abundance in Δ*sas6l* parasites, suggesting that compensation through transcriptional upregulation of this homologue did not occur. Moreover, genes such as *isp1*, *isp3*, *ppkl*, *nek2* and *nek4*, which have been showed to be involved in female/zygote/ookinete development^29^,^30^,^31^,^32^, showed no significant difference in transcription in activated gametocytes of the Δ*sas6l* parasites (Fig. 5e). Finally, to assess whether *sas6l* transcripts are repressed during early stages of zygote development by the DDX6-class RNA helicase DOZI required for translational repression^33^, we analysed mRNA expression of *sas6l* in a ∆*dozi* mutant and showed that expression of *sas6l* compared to WT was not significantly decreased in activated gametocytes (Fig. 5f).

## Discussion

The flagellum protein SAS6 is required for centriolar organisation and spindle assembly, and has been shown to be crucial, not only to *Plasmodium* transmission through the sexual stages, but for the formation of flagella in a range of species^1^. The SAS6 paralogue, SAS6L, however, has been more intriguing with respect to its location and function in diverse eukaryotes^3^. While it is associated with the flagellum in trypanosomatids, it is associated with the conoid of the apical complex in *T. gondii*, adding weight to the hypothesis that this divergent structure in apicomplexans has some evolutionary link to flagellar structures^3^,^26^. Gene deletions in these taxa suggest that SAS6L is not essential to cell viability, unlike SAS6. Nevertheless, SAS6L presents a possible marker of the conoid-associated structures in Apicomplexa.

The apparent lack of a conoid in *Plasmodium*, and the unusual formation of the flagella axonemes within the cytosol prior to exflagellation of male microgametes, suggested that SAS6L might function in flagellum assembly in *Plasmodium*^3^. We, however, observed that SAS6L is absent during microgamete development, and is instead located to the apical end of the two invasive stages of *Plasmodium* that develop within the mosquito; ookinetes and sporozoites. Detailed analysis of the apical tip localisation in these two *Plasmodium* cell stages showed for the first time that SAS6L occurs as a ring structure, and we confirmed that SAS6L presents as a similar ring in *Toxoplasma* tachyzoites. Occurrence as a ring also suggests some conserved functional/structural capability shared with SAS6 where the globular domain is also responsible for forming a ring structure. Indeed, over expression of SAS6L in *Toxoplasma* caused excess filament assembly, suggesting that these assembly properties of SAS6L remain^3^.

SAS6L is expressed early in ookinete (<4 h) and sporozoite (<14 dpi) development, consistent with the qRT-PCR data that shows that mRNA is already upregulated in activated gametocytes presumably ready for translation in early macrogamete stages. Establishment of cell polarity, a crucial step in the formation of new apicomplexan cells, is thought to begin from the apical end, and a number of IMC, apical complex and conoid ring proteins are implicated in this process^17^,^29^,^34^,^35^. The early expression of SAS6L indicates that it is associated with these early stages of development of cell polarity. The initially cytosolic distribution of SAS6L in zygotes and early oocysts, before it resolves to the developing apical complex, suggests that it is awaiting a cue from some other assembly process before forming an apical end localisation. It is unknown whether all of the cytosolic SAS6L is incorporated into the nascent apical complex or whether some of it is degraded upon zoite maturation.

It is intriguing that *Toxoplasma* presents a very similar pattern of SAS6L behaviour during daughter cell assembly, with expression of cytosolic protein before its incorporation into developing daughter apices. The timing of conoid formation in *Toxoplasma* is not precisely known, however bleaching experiments of tubulin-YFP shows that incorporation of conoid tubulin occurs after the initial development of daughter pellicles^16^. The timing of accumulation of SAS6L to the daughter apex is similar, and it is possible that SAS6L relocation to the apex is dependent on conoid assembly. In any case, it is striking that SAS6L behaviour bears strong similarities during nascent apical complex formation in both *Plasmodium* and *Toxoplasma*.

The differential expression of SAS6L in the apical complex of ookinetes and sporozoites, compared to merozoites in the mammalian liver (hepatocyte) and blood stages provides evidence that there is differentiation of the apical complex between the different invasive forms of *Plasmodium.* Evidence for compositional differences suggests adaptation to different requirements of these different invasion events. This might reflect properties of the cell types being invaded (e.g. erythrocytes verses mosquito gut cells), the number and diversity of invasion/transgression events required per zoite (e.g. individual sporozoites must invade the mosquito salivary glands as well as mammalian liver cells), or the demand for successful invasion events (e.g. small numbers of sporozoites injected into the mammalian host demand high invasion success rates for effective host transmission). There are several examples where the composition of the apical complex is different between invasive stages, for example: CelTOS, which has a specific role in parasite invasion^36^; and several alveolins, required for structure and motility^37^,^38^. In the case of alveolins additional paralogues may substitute at different life cycle stages^39^, however no other SAS6L paralogues have been identified. Therefore, the loss of SAS6L from the apical complex of merozoites, and ultrastructural evidence for the simplicity of its apical complex, suggests that this stage has become more minimal, possibly as a consequence of high parasite numbers and specialisation in erythrocyte invasion.

*Plasmodium* has been traditionally considered to lack a conoid based on ultrastructural data, (notably often from the blood-stage merozoites). Hence its classification in the Aconoidasida along with piroplasms (e.g. *Theileria* and *Babesia*) that are also considered to lack conoids^40^. However, some ultrastructural studies of *Plasmodium*, and other Hematozoa, do report additional circular/conical structures within the apical complex^22^,^41^. Currently our definition of the conoid is limited to ultrastructural, in particular the conspicuous tubulin-based barrel or collar structure with further rings at its apical end, as seen in taxa such as coccidians. However, we know that many other proteins associate with the conoid, and it is unknown what the functional consequence of the reduction of the tubulin structure alone would be. The presence of genes for SAS6L, throughout “Aconoidasida” lineages including *Theileria* and *Babesia* (Supplementary Fig. S3), therefore provide cause to revise our understanding of the extent of conoid reduction/loss in these groups.

Here, we showed that a flagellum-derived protein, shown previously to associate directly with the conoid of *Toxoplasma*, is found at the apical end of two invasive extracellular stages of *Plasmodium*. Its similar localisation and ring-like structure suggest a conserved function between *Toxoplasma* and *Plasmodium*, and possibly throughout Apicomplexa where this gene occurs. The lack of an observable phenotype in *∆sas6l Plasmodium* parasites is surprising given the early assembly during apical complex formation, and its apparent self-assembly properties that suggest a structural role. However, this is consistent with only a very subtle growth phenotype seen when *sas6l* was deleted in *T. gondii*, leaving the functional role of SAS6L presently obscure^3^. Its conservation across apicomplexan diversity strongly suggests it does have a significant role, and given that SAS6L is associated with the conoid in *T. gondii*, it implies that some functionality of the conoid persists in many taxa that are generally considered to lack a conoid, including mosquito-stage zoites of *Plasmodium* spp.

## Material and Methods

### Ethics statement

All animal work has passed an ethical review process and was approved by the United Kingdom Home Office. Work was carried out in accordance with the United Kingdom ‘Animals (Scientific Procedures) Act 1986’ and in compliance with ‘European Directive 86/609/EEC’ for the protection of animals used for experimental purposes under UK Home Office Project Licenses (40/3344 and 30/3248).

### Animals

Six-to-eight week old female Tuck-Ordinary (TO) (Harlan) outbred mice were used for all experiments.

### Generation of transgenic parasites

For GFP-tagging of *P. berghei sas6l* by single homologous recombination, a 1007 bp region of *sas6l* (PBANKA_141490) starting 0.5 kb downstream of the ATG start codon and omitting the stop codon was amplified using primers T1741 and T1742. This was inserted upstream of the *gfp* sequence in the p277 vector using *Kpn*I and *Apa*I restriction sites. The p277 vector contains the human *dhfr* cassette, conveying resistance to pyrimethamine. Before transfection, the vector was linearised with *PacI*. The gene knockout targeting vector for ∆*sas6l* was constructed using the pBS-DHFR plasmid, which contains polylinker sites flanking a *T. gondii dhfr/ts* expression cassette conveying resistance to pyrimethamine, as described previously^42^. PCR primers N0901 and N0902 were used to generate a 585 bp fragment at the 5′ end of the *sas6l* sequence from genomic DNA, which was inserted into *Apa*I and *Hind*III restriction sites upstream of the *dhfr/ts* cassette of pBS-DHFR. A 712 bp fragment generated with primers N0903 and N0904 from the 3′ region of *sas6l* was then inserted downstream of the *dhfr/ts* cassette using *Eco*RI and *Xba*I restriction sites. The linear targeting sequence was released using *Apa*I/*Xba*I.

All of the oligonucleotides used to make these constructs can be found in Supplementary Table S1. *P. berghei* ANKA line 2.34 (for GFP-tagging) or ANKA line 507cl1 (for gene deletion) were then transfected by electroporation^28^. Briefly, electroporated parasites were mixed immediately with 100 μl of reticulocyte-rich blood from a phenylhydrazine (6 mg/ml) (Sigma) treated, naïve mouse, incubated at 37 °C for 20 min and then injected intraperitoneally. From day 1 post infection pyrimethamine (70 μg/ml) (Sigma) was supplied in the drinking water for four days. Mice were monitored for 15 days and drug selection was repeated after passage into a second mouse. Resistant parasites were then cloned by limiting dilution (knockout) and genotyped.

For reporter protein tagging of SAS6L in *T. gondii*, single homologous recombination was again used. We amplified 973 bp immediately upstream of the *sas6l* (TGME49_301420) stop codon using RHDHXGPRT genomic DNA and primers Tgsas6l FW and Tgsas6l RV (Supplementary Table S1). Golden Gate modular assembly^43^ was used to in-frame fuse this to the coding sequence for HA-APEX (hemagglutinin epitope fused to an engineered ascorbate peroxidase: see Supplementary Fig. S1) in a plasmid containing the chloramphenicol-resistance gene^17^. This plasmid was linearised within the *sas6l* gene and transfected into parasites for integration and selection as previously described^17^. Cloned parasite lines were used for microscopic studies.

### Genotypic analysis of mutants

For the C-terminal fusion GFP tagged *P. berghei* parasites, diagnostic PCR was used as shown in Supplementary Fig. S1. Primer 1 (IntT1741) and primer 2 (ol492) were used to confirm integration of the GFP targeting construct (Supplementary Table S1). Parasites were visualised using a Zeiss AxioImager M2 (Carl Zeiss, Inc) microscope fitted with an AxioCam ICc1 digital camera (Carl Zeiss, Inc) and analysed by Western blot to confirm GFP expression and the correct protein size.

For the gene knockout parasites, diagnostic PCR was used as shown in Supplementary Fig. S2. Primer 1 (IntN90) and primer 2 (248) were used to determine if successful integration of the targeting construct had occurred. Primer 3 (N90KO1) and primer 4 (N90KO2) were used to confirm deletion of the *sas6l* gene. Two independent clones from two independent transfections were used for the phenotypic analyses.

### Phenotypic analysis

Exflagellation was examined on day 4 to 5 post-infection using ookinete culture medium^44^. After 15 min exflagellation centres were counted by phase contrast microscopy. Ookinete formation was assessed the next day. Cultures were pelleted for 2 min at 5000 rpm and then incubated with 50 μl of ookinete medium containing Hoechst 33342 DNA dye to a final concentration of 5 μg/ml and a Cy3-conjugated mouse monoclonal antibody 13.1^45^ recognising the P28 protein on the surface of ookinetes and any undifferentiated macrogametes or zygotes. P28-positive cells were counted with a Zeiss AxioImager M2 microscope (Carl Zeiss, Inc) fitted with an AxioCam ICc1 digital camera. Ookinete conversion was expressed as the percentage of P28 positive parasites that had differentiated into ookinetes^46^.

For mosquito transmission experiments and GFP localisation, 20–50 *Anopheles stephensi* SD500 female mosquitoes were allowed to feed for 20 min on anaesthetised infected mice whose asexual parasitaemia had reached ~12–15% and were carrying comparable numbers of gametocytes as determined by Giemsa stained blood films. 14 and 21 days post-infection (dpi) 20 mosquitoes were dissected and oocysts on their midguts counted. Oocyst formation was examined following Hoechst 33342 staining in PBS for 10–15 min and guts were mounted under Vaseline-rimmed cover slips. Counting was performed and images were recorded using 10x and 63x oil immersion objectives on a Zeiss AxioImager M2 microscope fitted with an AxioCam ICc1 digital camera. At 14 and 21 dpi, the same mosquito midguts used to record the oocyst number were homogenised in a loosely fitting homogeniser to release sporozoites, which were then quantified using a haemocytometer. Only for 21 dpi mosquitoes, salivary glands were dissected and homogenised in a loosely fitting homogeniser to release sporozoites, which were then quantified using a haemocytometer. Mosquitoes infected with WT or Δ*sas6l* parasites were used to perform bite back experiments with a TO mouse. Parasitemia was measured for WT and Δ*sas6l* by Giemsa staining at 4 dpi. Since both Δ*sas6l* clones gave similar phenotypic results, we analysed all of the data together.

### Purification of stage specific parasites

Parasite stages were isolated as discussed previously^47^. For schizont cultures, blood cells from day 5 p.i. mice were placed in culture for 24 h at 37 °C (with rotation at 100 rpm) and separated from the uninfected erythrocytes on a 60% NycoDenz gradient (27.6% w/v NycoDenz in 5 mM Tris-HCl, pH 7.20, 3 mM KCl, 0.3 mM EDTA). Schizonts were harvested from the interface. Non-activated gametocytes (NAG) were isolated from day 4 to 5 post-infection blood kept on ice. Gametocytes were separated from uninfected erythrocytes on a 48% NycoDenz gradient in fresh coelenterazine loading buffer (CLB; PBS, 20 mM HEPES, 20 mM Glucose, 4 mM sodium bicarbonate, 1 mM EGTA, 0.1% w/v bovine serum albumin, pH 7.25). Gametocytes were harvested from the interface and washed twice in RPMI 1640. Activated gametocytes (AG) were then incubated with ookinete culture medium^44^ for 30 min at 20 °C. For ookinete samples, infected blood cells from day 5 p.i. mice were pelleted for 2 min at 5000 rpm and then incubated with 50 μl of ookinete medium for 24 h. Ookinetes were purified on a 63% NycoDenz gradient, harvested from the interface and washed. On 14 and 21 dpi, sporozoites were isolated from 20 *Anopheles stephensi* SD500 female mosquitoes which were previously fed for 20 min on anaesthetised infected mice whose asexual parasitaemia had reached ~12–15%. Mosquitoes were dissected and oocysts/salivary glands washed in PBS.

### Western blotting

Activated gametocyte and ookinete samples were isolated as described below. WT or SAS6L-GFP samples were then purified using a GFP-Trap kit to immunoprecipitate GFP-fusion protein (Chromotek). After the addition of Laemmli sample buffer, the samples were boiled and loaded on a 4–12% SDS-polyacrylamide gel. Samples were subsequently transferred to nitrocellulose membranes (Amersham Biosciences) with immunoblotting performed using the Western Breeze Chemiluminescent Anti-Rabbit kit (Invitrogen) and anti-GFP polyclonal antibody (Invitrogen) at a concentration of 1:1250, according to the manufacturer’s instructions.

### Quantitative RT-PCR

Total RNA was isolated from purified parasites using an RNeasy purification kit (Qiagen) and cDNA was synthesised using an RNA-to-cDNA kit (Applied Biosystems). qRT-PCR used SYBR green fast master mix (Applied Biosystems) and analysis was conducted using an Applied Biosystems 7500 fast machine. WT expression was determined using the Pfaffl method^48^. Relative quantification in the mutant line was normalised against WT expression using the ∆∆Ct method. Both methods used a combination of *hsp70, seryl-tRNA synthetase* and *arginyl-tRNA synthetase* as reference genes. Three biological replicates were used for each experiment, which was repeated at least twice. See Supplementary Table S1 for a full list of the primers used for qRT-PCR. Statistical analyses were performed using Excel and GraphPad Prism (GraphPad Software). For relative gene expression, a Student’s unpaired *t*-test was used.

### Live imaging of parasites

Images were captured using a 63x oil immersion objective on a Zeiss AxioImager M2 microscope fitted with an AxioCam ICc1 digital camera and analysed with the AxioVision 4.8.2 software and prepared for publication using Adobe Photoshop. Ookinetes were stained with a Cy3-conjugated mouse monoclonal antibody 13.1 which recognises the P28 protein on the parasite surface^45^.

### Generation of dual tagged parasite lines

Parasites (gametocytes) that express SAS6L-GFP were mixed with parasites expressing a mCherry-tagged version of the apical protein IMC sub-compartment protein, ISP1 (ISP1-mCherry^29^) and incubated at 20 °C for 24 h in ookinete media. Images were taken as described for live cell imaging.

### Liver stage imaging

For *P. berghei* liver stage parasites, infected cells were fixed with 4% PFA in PBS for 20 min at room temperature (RT) and washed in PBS. −20 °C methanol was added and the cells incubated at −20 °C overnight in methanol. Cells were washed 3 times in PBS and blocked for 1 h in 10% FCS-PBS. The primary antibody was incubated at 1 in 1000 (rabbit anti-GFP, Invitrogen) in 10% FCS-PBS. Cells were washed in PBS and then incubated at 1 in 3000 in anti-rabbit AlexaFluor 488 (in 10% FCS-PBS). DNA was visualised by staining with 1 μg/ml DAPI (Sigma). Cells were washed three times in PBS and once in water and then mounted with Dako Fluorescent Mounting Medium (Dako).

### Immunofluorescence assay

IFAs for *P. berghei* were performed on poly-lysine slides where schizont and activated gametocytes had been previously fixed in paraformaldehyde 4% with 1×PBS (schizont) or Microtubule-stabilising buffer (MTSB) (activated gametocytes) for 30 min at RT and smeared onto slides. Gametocytes were harvested as above and washed twice in RPMI 1640 ready for activation of gamete formation during 40 min before fixation. Schizonts were harvested from the interface and washed twice with 1×PBS before fixation. For *T. gondii*, parasites infecting adherent HFF cells on coverslips were similarly fixed in paraformaldehyde. The fixed cells were permeabilized with PBS containing 0.15% Triton X-100 for 10 min and washed three times with 1×PBS before saturation. Saturation was performed by using BSA 3% in 1×PBS during 1 h at RT and BSA 1% in 1×PBS was used to incubate antibodies into. Anti-GFP rabbit antibody was used at a dilution (1:250) (Invitrogen), anti-αtubulin mouse was used at a dilution (1:1000) (Sigma), anti-centrin mouse clone 20h5 was used at a dilution (1:500), and anti-IMC1 mouse used at dilution (1:500) (Ward lab), and each was incubated for 1 h at RT. Three washes were performed with 1×PBS then, AlexaFluor 488 labelled anti-rabbit (green) and AlexaFluor 568 labelled anti-mouse (red) (Invitrogen) (1∶1000) were used as a secondary antibodies and incubated for 1 h at RT. Slides were mounted with vectashield containing DAPI (blue) and sealed with nail polish. *P. bergehi* images were captured as described for live imaging using a 100x oil immersion objective, and *T. gondii* images were captured using a Nikon Ti-E widefield inverted microscope with a Hamamatsu Orca-Flash4.0 CMOS camera. Control images using a WT parasite line that does not express GFP were also taken using the same method to show background noise.

### Super resolution Imaging

To prepare imaging slides, coverslips of thickness No. 1.5H (0.170 mm ± 0.005 mm) were flamed and smeared with 0.1% Polyethylenimine (PEI) solution. Purified ookinetes or salivary gland sporozoites were fixed with 2%PFA in 1×PBS suspension and allowed to settle onto PEI-treated coverslips for 20 minutes and then probed with indicated antibodies (13.1 mouse monoclonal antibody for ookinetes and CSP antibody for sporozoites) by IFA, stained with DAPI, rinsed in water and mounted onto Vectashield on glass slides before sealing with nail varnish. Super-resolution images were acquired using a Deltavision OMX 3D-SIM System V3 BLAZE from Applied Precision (a GE Healthcare company) equipped with 3 sCMOS cameras, 405, 488, 592.5 nm diode laser illumination, an Olympus Plan Apo N 60 × 1.42NA oil objective, and standard excitation and emission filter sets. Imaging of each channel was done sequentially using three angles and five phase shifts of the illumination pattern as described previously^49^. The refractive index of the immersion oil (Cargille) was adjusted to 1.516 to minimize spherical aberrations. Sections were acquired at 0.125 μm z steps. Raw OMX data was reconstructed and channel registered in SoftWoRx software version 6.1.3 (Applied Precision, a GE Healthcare company).

## Additional Information

**How to cite this article**: Wall, R. J. *et al*. SAS6-like protein in *Plasmodium* indicates that conoid-associated apical complex proteins persist in invasive stages within the mosquito vector. *Sci. Rep.* **6**, 28604; doi: 10.1038/srep28604 (2016).

## Supplementary Material

Supplementary Information
