# Supplementary material for: SAS6-like protein in Plasmodium indicates that conoid-associated apical complex proteins persist in invasive stages within the mosquito vector
Source: Sci Rep. 2016 Jun 24;6:28604. doi: 10.1038/srep28604 (PMC4919640; doi:10.1038/srep28604)
Supplement: Supplementary Information [file srep28604-s1.pdf]

# SAS6-like protein in *Plasmodium* indicates that conoid-associated apical complex proteins persist in invasive stages within the mosquito vector

Richard J. Wall[1,#], Magali Roques[1,+], Nicholas J. Katris[2,3,+], Ludek Koreny[2,+], Rebecca R. Stanway[4], Declan Brady[1], Ross F. Waller[2,*] and Rita Tewari[1,*]

[1]School of Life Sciences, Queens Medical Centre, University of Nottingham, Nottingham, UK.
[2]Department of Biochemistry, University of Cambridge, Cambridge, UK
[3]School of Botany, University of Melbourne, Parkville, Victoria, Australia
[4]Institute of Cell Biology, University of Bern, CH-3012 Bern, Switzerland

[#]Current address: Division of Biological Chemistry and Drug Discovery, School of Life Sciences, University of Dundee, Dundee, UK

+ These three authors contributed equally
*Corresponding authors

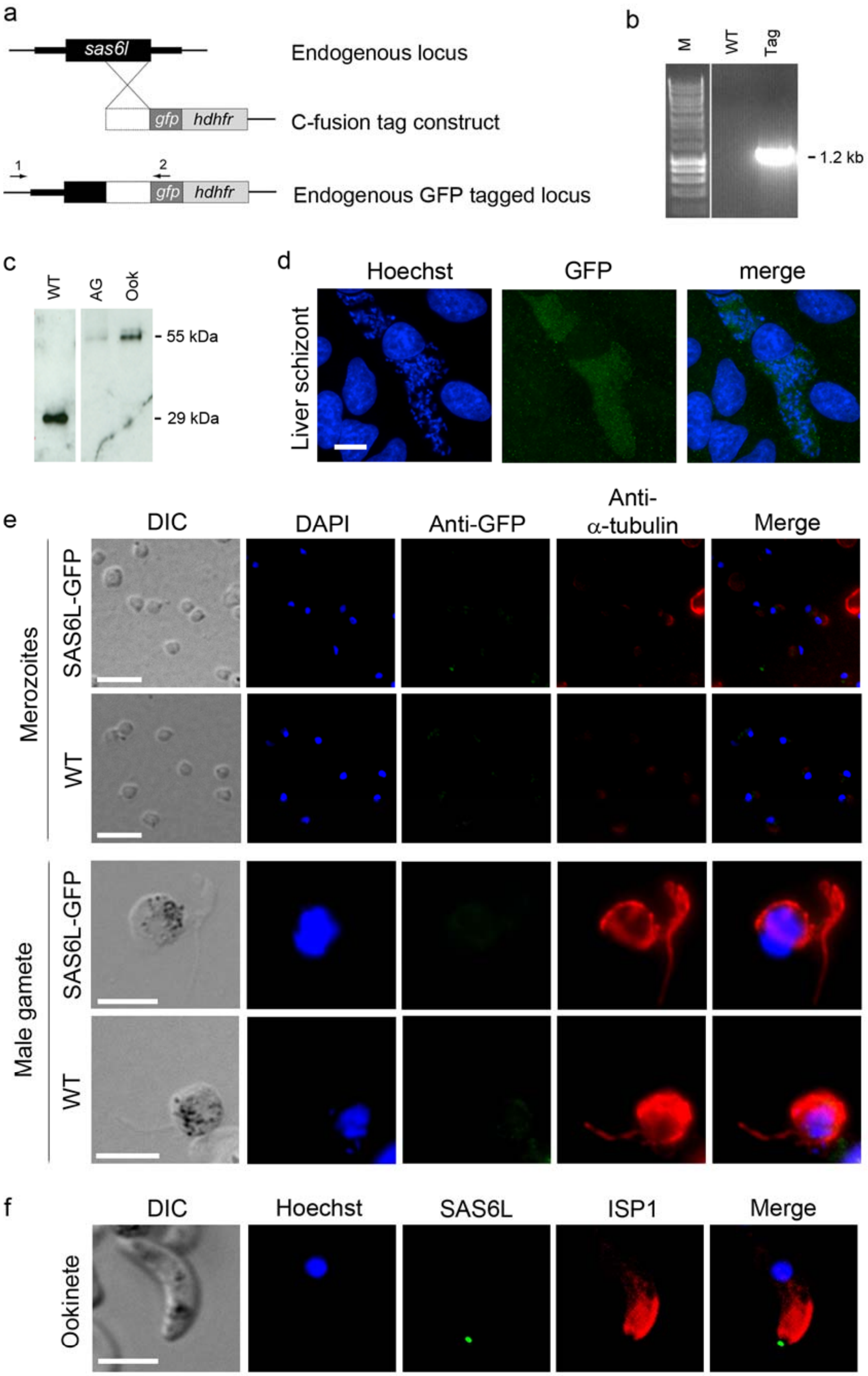


g

>HA-APEX
MGYPYDVPDYAGKSYPTVSADYQDAVEKAKKKLRGFIAEKRCAPLMLRLAFH
SAGTFDKGTKTGGPFGTIKHPAELAHSANNGLDIAVRLLEPLKAEFPILSYADF
YQLAGVVAVEVTGGPKVPFHPGREDKPEPPPEGRLPDATKGSDHLRDVFGK
AMGLTDQDIVALSGGHTIGAAHKERSGFEGPWTSNPLIFDNSYFTELLSGEKE
GLLQLPSDKALLSDPVFRPLVDKYAADEDAFFADYAEAHQKLSELGFADA

**Supplementary Fig. S1: Generation, genotypic analysis and immunofluorescence images of SAS6L-GFP parasite line**

(**a**) Schematic representation of the endogenous *sas6l* locus, the GFP-tagging construct and the recombined *sas6l* locus following single homologous recombination. Arrows 1 and 2 indicate the PCR primers used to confirm successful integration of the construct. (**b**) Diagnostic PCR of SAS6L-GFP and WT parasites using primers IntT174 (Arrow 1) and ol492 (Arrow 2). Integration of *the sas6l* tagging construct gives a band of 1.2 kb. TAG = SAS6L-GFP parasite line. (**c**) Western blot after GFP-Trap of SAS6L-GFP (55 kDa) and WT-GFP (29 kDa) protein to illustrate SAS6L-GFP in activated gametocytes (AG) and ookinetes (Ook) parasite stages. (**d**) Live fluorescent imaging of 54 hrs liver stage mature schizont with merozoites using the SAS6L-GPF parasite line. Nuclei were detected using Hoechst 33342 (blue). Merge is the composite of Hoechst and GFP signals. Scale bar = 10 μm. (**e**) Immunofluorescence images of blood stage merozoites and a male gamete for SAS6L-GFP and WT (not expressing GFP in any form) parasite lines. Green channel represents the anti-GFP antibody staining, red channel represents the anti-α-tubulin antibody staining. Nuclei were detected using DAPI in the vectashield mounting media (blue), and the cells were displayed by differential interference contrast (DIC). Merge is the composite of DAPI, anti-GFP and anti-α-tubulin antibodies. (**f**) Live imaging of dual tagged ookinetes using SAS6L-GFP (green channel) and ISP1-mCherry (red channel) parasite lines. Nuclei were detected using Hoechst 33342 (blue), and the cells were displayed by DIC. Merge is the composite of Hoechst, GFP and mCherry. Scale bars = 5 μm. (**g**) Protein sequence the C-terminal fusion HA-APEX (hemagglutinin epitope fused to an engineered ascorbate peroxidase) tag appended to *T. gondii* SAS6L.

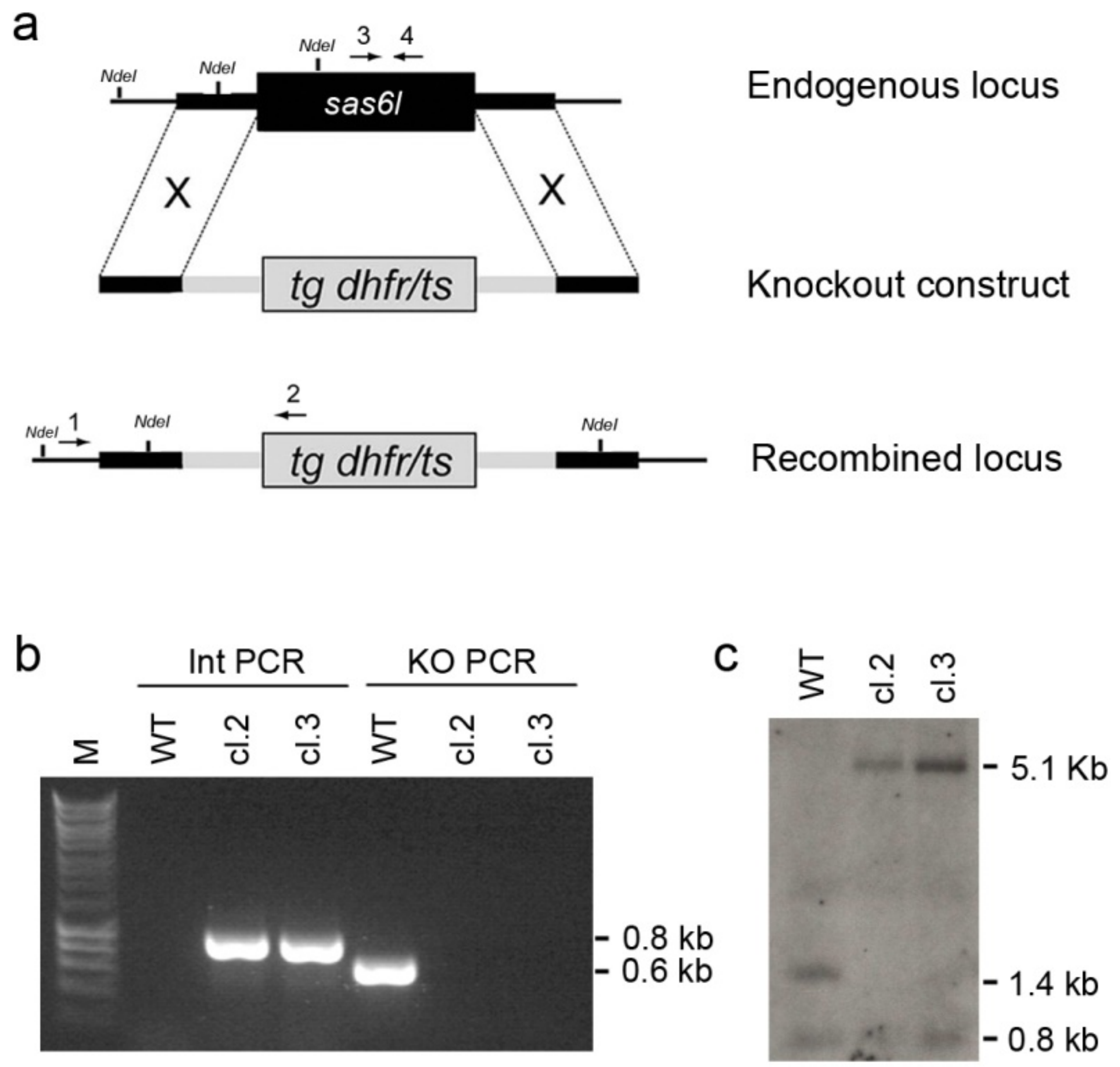


**Supplementary Fig. S2: Generation and genotypic analysis of ∆*sas6l* mutant parasite line**

(**a**) Schematic representation of the endogenous *sas6l* locus, the targeting gene deletion construct and the recombined *sas6l* locus following double homologous recombination. Arrows 1 and 2 indicate PCR primers used to confirm successful integration of the construct and arrows 3 and 4 indicate PCR primers used to confirm deletion of the *sas6l* gene in both clones. (**b**) Diagnostic PCR of the *sas6l* locus in ∆*sas6l* and WT parasites using primers IntN90 (Arrow 1) and ol248 (Arrow 2). Integration of the targeting construct gives a band of 0.8 kb. Presence of the gene gives a band of 0.6 kb (Arrows 3 and 4; N90KO1 and 2). (**c**) Southern blot analysis of ∆*sas6l* and WT parasite genomic DNA following *Nde*I digestion. A probe specific for the *sas6l* 5'UTR bound to a 1.4 kb band in WT, to a 5.1 kb band in ∆*sas6l* parasites and to a 0.8 kb band in all parasite lines. Data for ∆*sas6l* clone 2 and 3 (cl.2/3) are shown.

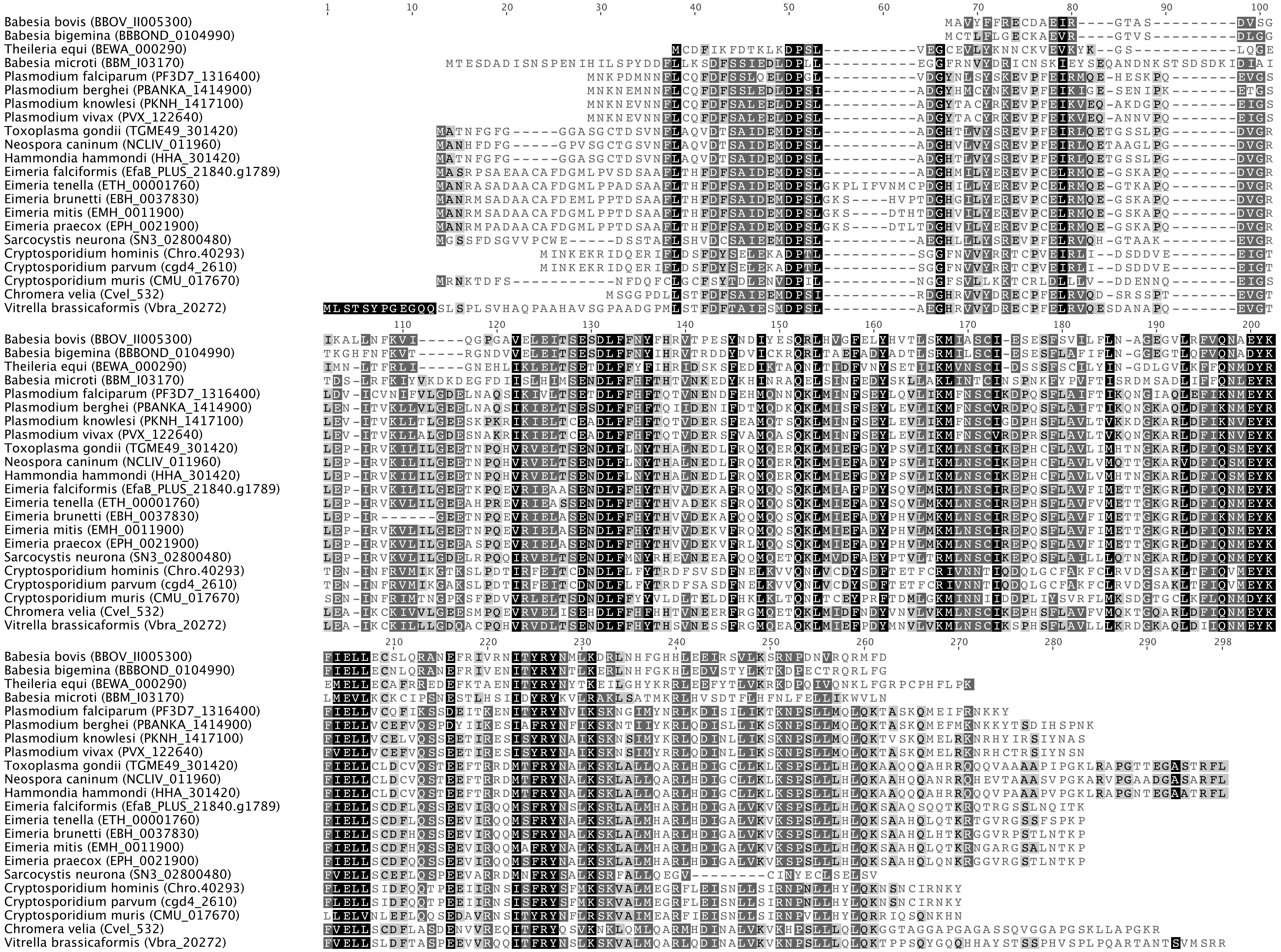
Babesia bovis (BBOV_II005300)
Babesia bigemina (BBBOND_0104990)
Theileria equi (BEWA_000290)
Babesia microti (BBM_I03170)
Plasmodium falciparum (PF3D7_1316400)
Plasmodium berghei (PBANKA_1414900)
Plasmodium knowlesi (PKNH_1417100)
Plasmodium vivax (PVX_122640)
Toxoplasma gondii (TGME49_301420)
Neospora caninum (NCLIV_011960)
Hammondia hammondi (HHA_301420)
Eimeria falciformis (EfaB_PLUS_21840.g1789)
Eimeria tenella (ETH_00001760)
Eimeria brunetti (EBH_0037830)
Eimeria mitis (EMH_0011900)
Eimeria praecox (EPH_0021900)
Sarcocystis neurona (SN3_02800480)
Cryptosporidium hominis (Chro.40293)
Cryptosporidium parvum (cgd4_2610)
Cryptosporidium muris (CMU_017670)
Chromera velia (Cvel_532)
Vitrella brassicaformis (Vbra_20272)

**Supplementary Figure S3: Alignment of select SAS6L proteins from apicomplexans and chromerids.**

**Supplementary Table S1: Oligonucleotides used in this study**

| Name | Sequence 5'-->3' | Notes |
|---|---|---|
| T1741 | CCCCGGTACCGAGCTAACTAGCGAATCCGATTTATTC | KpnI site underlined |
| T1742 | CCCCGGGCCCCTTATTTGGGCTGTGTATATCACTTG | ApaI site underlined |
| IntT174 | GGTGAGGAATTAAATGCACAAAG | |
| ol492 | ACGCTGAACTTGTGGCCG | |
| | | |
| N0901 | CCCCGGGCCCGGAAGATTTAGATCCATCAATAG | ApaI site underlined |
| N0902 | GGGGAAGCTTGTCATTTTTGATCTAAGATG | HindIII site underlined |
| N0903 | CCCCGAATTCGCAACTTCAAAAAACAGC | EcoRI site underlined |
| N0904 | GGGGTCTAGAGGCAAAACAAACTCTTCGCAAC | XbaI site underlined |
| IntN90 | GGTAATAATAAGGTTGAATAAAATTG | |
| ol248 | GATGTGTTATGTGATTAATTCATACAC | |
| N90 ko1 | GTGTATATATTTATTTCCATTTTTTAG | |
| N90 ko2 | CCGGGGATTGAACAAATTCACACAC | |
| | | |
| hsp70 FW | GTATTATTAATGAACCCACCGCT | PBANKA_081890 |
| hsp70 RV | GAAACATCAAATGTACCACCTCC | |
| arginyl-tRNA FW | TTGATTCATGTTGGATTTGGCT | PBANKA_143420 |
| arginyl-tRNA RV | ATCCTTCTTTGCCCTTTCAG | |
| seryl-tRNA FW | CAACCGCTATGCGCATTACAC | PBANKA_061540 |
| seryl-tRNA RV | CTCAACCTTATCAAACTGATGAACT | |
| nek2 FW | AGAGGCATTTATTGAAGACGG | PBANKA_124070 |
| nek2 RV | GCTGTAATTATCTGTAGCAACCA | |
| nek4 FW | GATGTATGGGCTATTGGCT | PBANKA_061670 |
| nek4 RV | AGCATAACTGTTGAATTCCCT | |
| isp1 FW | GCCACCAAAAGGTACGAATG | PBANKA_120940 |
| isp1 RV | GCCAAACAACAATTGCCACT | |
| isp3 FW | AGCTTGTGCTGCATTAACGA | PBANKA_132430 |
| isp3 RV | TTGAATTTCATTTCCATCAGGA | |
| ppkl FW | TTCTAAAGTACCTTCACCAAGAG | PBANKA_132950 |
| ppkl RV | TAGCAGGTCCTTCTTTACAC | |
| dozi FW | GCAAGAATGTCGCAAACAC | PBANKA_121770 |
| dozi RV | TCTGAGGAAACTAAACATCGAC | |
| sas6 FW | GAACAATATCACTGCATCCCC | PBANKA_010620 |
| sas6 RV | GCTGGTGTGTAACCTATTCCT | |
| sas6l FW | GTAAAATTATTAGTTCTAGGTGAGGAATT | PBANKA_141490 |
| sas6l RV | GTTTGTCTTGCATTGTATCGAATATG | |
| Tgsas6l FW | TGGTCTCAGGAGCTTCTGCTCTTGTTCTGCGTGTATTTC | TGME_49301420 |
| Tgsas6l RV | TGGTCTCACGAAAGGAACCGAGTGGATGC | |
